# Silver diamine fluoride with sodium fluoride varnish versus silver diamine fluoride in arresting early childhood caries: a 6-months follow up of a randomized field trial

**DOI:** 10.1186/s12903-023-03597-5

**Published:** 2023-11-17

**Authors:** Enas B. Abdellatif, Mona K. El Kashlan, Maha El Tantawi

**Affiliations:** Department of Pediatric Dentistry and Dental Public Health, Faculty of Dentistry, Alexandria University, Champolion St, Azarita, 21527 Alexandria Egypt

**Keywords:** Early childhood caries, Silver diamine fluoride, Sodium fluoride varnish, Children, Enamel caries, Dentin caries, Primary teeth, Rural

## Abstract

**Background:**

Early childhood caries (ECC) is the most prevalent chronic health problem in young children, and it can be arrested using professionally applied fluoride such as Sodium fluoride (NaF) varnish and Silver Diamine Fluoride (SDF). This trial compared two interventions to arrest ECC lesions: 38% SDF combined with 5% NaF varnish versus 38% SDF and assessed whether the arrest rate was affected by baseline lesion severity measured by ICDAS.

**Methods:**

Children aged ≤ 4 years from 4 nurseries in a rural area in Alexandria, Egypt joined the study in March 2022. They were included if they had at least one active carious lesion with ICDAS codes ≥ 3. They were randomized to receive either 38% SDF with 5% NaF varnish or 38% SDF alone. In both groups, the agents were applied at baseline and after 6 months on the caries lesions. NaF was additionally applied on all teeth in the oral cavity, and it was also applied after three months. The primary outcome was lesion arrest status after six months. Parents’ satisfaction with their children’s appearance was the secondary outcome. Pearson Chi-Square test was used for bivariate comparison and multi-level multiple logistic regression was used to assess the effect of the intervention on caries arrest controlling for confounders. The interaction between the intervention and baseline lesion severity (categorized into moderate and severe lesions) was assessed and the p value was calculated.

**Results:**

The study included 1606 lesions in 220 children, median (IQR) age = 48(9) months. The percentages of arrested lesions after the application of SDF + NaF and SDF only were 77.7% and 73.2% (p = 0.035). In multivariable analysis, SDF + NaF had significantly greater caries arrest effect than SDF alone (AOR = 2.12, p = 0.03) with significant difference (p = 0.03) between moderate (AOR = 4.10, p = 0.005) and advanced (AOR = 1.92, p = 0.08) lesions. Most parents were satisfied with their children’s appearance with no significant difference between groups (SDF + NaF = 84.5%, SDF = 78.18%, p = 0.23).

**Conclusion:**

SDF + NaF had a higher arrest rate than SDF alone and this difference was significant in moderate but not advanced lesions. The findings have implications for the non-invasive management of ECC.

**Trial registration:**

This trial was registered in the clinicaltrials.gov registry (#NCT05642494).

## Introduction

Early childhood caries (ECC), defined as any carious, filled, or missing tooth in children younger than 6 years of age [1], is the most prevalent chronic health problem in young children, beginning early in life and progressing more rapidly than caries in adults [2]. Untreated caries in deciduous teeth was reported to be the 10th most prevalent condition globally in 2017, affecting 532 million children worldwide [3]. Untreated decay in primary teeth leads to pain, sepsis, spread of infection and malnutrition due to inability to eat and, thus, poor health [1]. Also, ECC has a negative impact on the quality of life, growth, social development, neurodevelopment, and wellbeing of affected children [4]. Children affected by ECC may need treatment under general anesthesia [5] with high risk for children, economic burden for families and great cost for healthcare systems [6].

ECC can be controlled by the professional application of fluoridated agents such as fluoride varnish and Silver Diamine Fluoride (SDF). These fluoride agents can slow the progress of ECC lesions, especially at the initial stages and reduce the cost of treatment by decreasing the need for advanced and complex treatment, in addition to reducing the number of treatment visits and the need for general anesthesia. [7]. NaF varnish prevents caries, arrests enamel lesions and soft dentine caries [8]. According to a recent meta-analysis, the percentage of remineralised enamel caries was 63.6% when 5% NaF varnish was used [7]. SDF remineralises carious dentin by forming a layer rich in calcium and phosphate around the carious lesion [9] and inhibiting the degradation of dentin organic matrix [10]. Systematic reviews confirm the effectiveness of SDF in arresting dentin caries in primary teeth, with success rates ranging from 65 to 91% [7, 11].

A superior caries arrest effect was reported for SDF over NaF, especially for dentine caries in primary teeth [12]. SDF also had greater effectiveness than other active treatments or placebo agents [13]. SDF is easy to apply and offers a minimally invasive treatment option [14]. Thus, managing caries with SDF may be suitable for young, uncooperative, or socially vulnerable children [15]. However, one of the main drawbacks of SDF is staining of teeth which may affect its acceptability to parents although this acceptability may differ from one setting to another based on cultural norms and expectations [15].

NaF varnish can remineralise enamel caries and SDF is effective in arresting dentine caries [7, 15].Thus, adding both agents together may increase the caries arrest rate by tackling caries lesions at different stages of progression. Adding NaF over SDF may also allow SDF to be in contact with the carious lesion for as long as possible, prevent saliva from diluting the SDF, reduce the risk of staining other tooth surfaces, and mask the taste of SDF [16] with the possibility of greater caries arrest.

Egypt has an ECC problem. The latest national oral health survey showed that the prevalence of ECC was 69.2% [17]. Also, a global study reported that ECC prevalence exceeded 50% [18]. At the same time, the availability and accessibility of regular dental care for preschool children in rural settings, where 57% of Egyptians live, is limited [19]. Thus, simple, and inexpensive preventive modalities and minimally invasive procedures are needed to address the high burden of ECC in the country.

This trial compared two interventions to arrest ECC in children 4 years old and younger: 38% SDF combined with 5% NaF varnish versus 38% SDF solution. The trial also assessed whether the arrest rate was affected by baseline lesion severity, measured by the International Caries Detection and Assessment System (ICDAS II) [20], including moderate lesions (ICDAS code 3 or 4) and advanced lesions (ICDAS code 5 or 6). The null hypothesis was that there would be no significant difference in ECC arrest between the two interventions.

## Methods

### Design and ethical consideration

Ethical approval for this randomized, controlled, parallel-groups field trial was granted by the Research Ethics Committee of the Faculty of Dentistry, Alexandria University (#0274-07/2021). This trial was registered in the clinicaltrials.gov registry (#NCT05642494). The research procedures, risks and benefits were explained to the parents, and they were asked to sign an informed consent form. The study was conducted in accordance with the Helsinki declaration [21] and reported following the CONSORT guidelines [22]. Participants were referred for treatment for teeth showing ECC progress after the intervention.

### Participants, settings, and location

Children were invited to join the study if they were healthy, ≤ 4 years of age, with at least one active carious lesion on a primary tooth, with ICDAS codes 3 and higher as reported by Duangthip et al. [12]. Teeth with spontaneous or elicited pain due to caries, teeth showing any sign of pulpal infection, swelling and/or abscess were excluded. Also, children were excluded if they had allergies to silver or any material used in the study. All eligible teeth per child were included. The study was conducted in a rural area in Alexandria, Egypt. Children were recruited from 4 nurseries in 3 villages.

### Interventions

The children received one of two interventions with an allocation ratio = 1:1. The control group received 38% SDF solution (Advantage Arrest, Elevate Oral Care, FL, USA). Petroleum jelly was applied on the lips and perioral skin as a protective barrier to avoid staining. Gross debris was removed from the carious cavities to allow better SDF contact with denatured dentin. No attempt was made to remove carious tissues before SDF application. The areas to be treated were isolated with cotton rolls and dried with dry cotton pellets. One drop of SDF was carefully applied with a microbrush. Carious lesions were painted for 10 s, and the excess was removed using cotton pellets. The solution was left in contact with the tooth surface for one minute before the child was allowed to close their mouth [12]. The application was done at baseline and after 6 months [15].

The test group received the same 38% SDF solution as the control group and 5% NaF varnish (ALPHA-PRO ® WHITE VARNISH, USA). After the application of SDF using the method described in the control group, it was left to dry for 1 min. Partial isolation of the whole oral cavity was done using cotton rolls. 0.5 ml of 5% NaF varnish was painted over the carious lesion for one minute and on all remaining teeth in the oral cavity for 2–4 min [23]. SDF + NaF were applied at baseline and after 6 months. The 5% NaF varnish alone was re-applied after 3 months from baseline [15]. After the application of the fluoride agents in both groups, the child was instructed not to drink or eat for at least 30 min.

### Outcome assessment

#### Primary outcome

The primary outcome was the arrest status of carious lesions after six months. Three examiners (EBA, MQ, and RY) were trained on assessing caries in two levels; The first level was based on caries experience presence (dmfs) following the WHO criteria. The second level was based on caries stage using the ICDAS criteria [20] and lesion activity using the ICDAS-lesion activity assessment (LAA) criteria [24]. The training was based on the online resources for ICDAS method of caries examination [25]. This was followed by assessing caries in 10 children for training and consensus building. The inter and intra examiner agreement levels of the three examiners were then checked by examining 15 other children not included in the study with a repeated examination after a week. The Kappa statistic for the inter and intra examiner agreement for assessing the presence of caries experience and caries stage using the ICDAS criteria ranged from 0.89 to 0.96 which denotes excellent agreement [26].

The oral examinations were conducted in the nurseries under natural daylight where children were seated in front of a window. No magnification or radiographs were used, and potential lesions were cleaned and dried by a piece of cotton before examination. Assessing lesion activity was done by visual inspection and tactile detection using a 0.5 mm ball-ended Community Periodontal Index (CPI) periodontal probe and a disposable dental mirror. Lesions were assessed at baseline and after 6 months. At baseline, lesions with ICDAS code 3 were recorded as active if the surface was whitish/ yellowish opaque with loss of luster; or felt rough when the tip of the probe was moved. All lesions with ICDAS code 4 were recorded as active. After 6 months, lesions coded as ICDAS 3 or 4 were classified as arrested if there was no progress to cavitation and the lesions did not become ICDAS 5 or 6 [12].

Lesions that were cavitated at baseline with ICDAS codes 5 or 6 were recorded as active if softness was detected upon gentle probing [27]. After 6 months, if the wall or floor of the lesion was soft and easily penetrated by the probe using light force, it was diagnosed as active. A lesion where all surfaces were hard and smooth was diagnosed as arrested [12, 27].

#### Secondary outcomes

After the application of the fluoride agents, parents were asked if they were satisfied with the appearance of their children’s teeth and the responses were recorded as satisfied or unsatisfied [28]. A week after applying the interventions, parents were asked about adverse effects of SDF including tooth or gum pain, gum swelling, gum bleaching, and systemic toxicity such as nausea, vomiting, or generalized discomfort [29]. Staining of the lesions was recorded as present after applying the interventions if any black stain appeared where the agents were applied or absent if no change was observed in lesion color [29].

### Confounders

In addition to assessing the ICDAS code and lesion activity, the clinical examination assessed oral hygiene using the plaque index (PI) of Silness and Loe on 6 index teeth (52, 55, 64, 72, 75, and 84) [30] and caries experience by recording the number of decayed, missing and filled surfaces (dmfs) using the World Health Organization (WHO) criteria [31].

The Arabic version of the WHO questionnaire for oral health assessment of children [32] was used to collect information by interviewing the mothers. The questionnaire assessed the child’s demographic characteristics including age in months, sex, and mother’s education (No formal schooling, Primary school completed, Middle school completed, High school, College/university completed). The questionnaire also assessed oral health behaviors such as toothbrushing (Never, Several times a month (2–3 times), Once a week, Several times a week (2–6 times), Once a day, 2 or more times a day), dental visits during the previous year (Once, Twice, Three times, Four times, More than four times, no visit during the past 12 months, never received dental care/visited a dentist, I don’t know/don’t remember) and sugar consumption (at least once daily versus less frequent consumption) of eight types of sugary products: fruit, biscuits and cakes, carbonated beverages, jam and honey, sugar-added chewing gums, candies, sugar-sweetened milk and sugar-sweetened hot drinks.

### Sample size determination

Sample size was estimated assuming 5% alpha error and 80% study power. Caries arrest rate after 6 months of a single application of 38% SDF was reported to be 43.9% [33]. After two applications of 5% NaF varnish, caries arrest rate after 12 months was calculated in a previous study to be 20.9% [27]. Assuming an additive effect of 2 applications of 5% NaF and 38% SDF, the caries arrest rate at 6 months in the SDF + NaF group was calculated to be 64.8%. The required number of children per group was 88 as calculated by G*power 3.0.10 [34]. Expecting a drop-out of 20%, the number of children to be recruited per group was estimated to be 106 ≈ 110. Total sample size = number of groups X number per group = 2 × 110 = 220 children.

### Randomization, allocation, and blinding

The children were equally allocated to the two groups via block randomization, using a computer-generated random sequence in blocks of 4 [35]. The allocation sequence was kept in opaque sealed envelopes by a trial independent individual (NN). The child was examined by an investigator (EBA) to confirm eligibility and conduct the clinical examination. The trial independent person (NN) opened the envelope and informed EBA of the allocated group to apply the intervention. The children and their families as well as the outcome assessors and biostatistician were blinded to the intervention type.

**Fig. 1:**
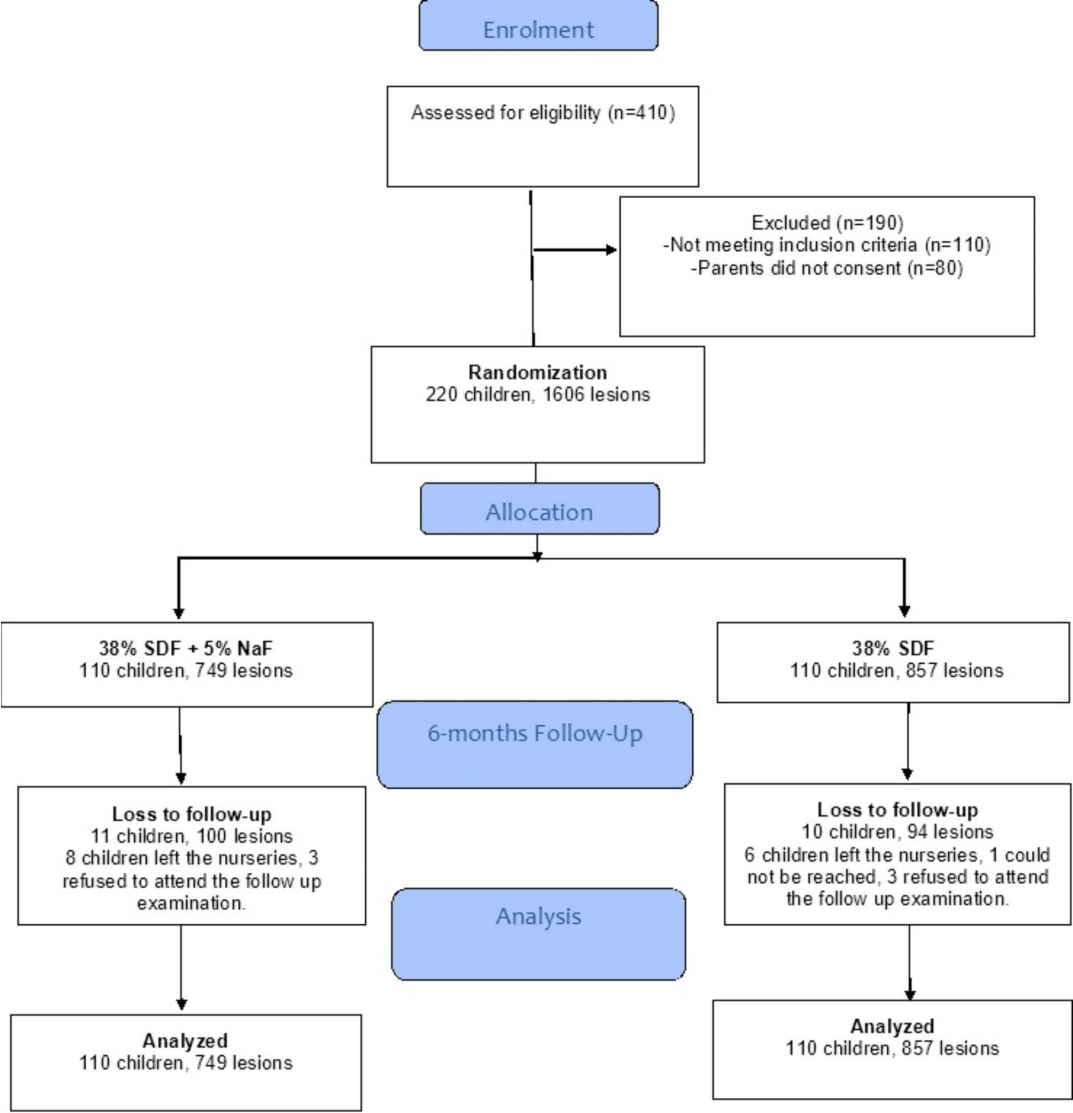
Flow of participants during the study period

### Statistical analysis

Data were analyzed using IBM SPSS Statistics for Windows, Version 23.0. Armonk, NY: IBM Corp and significance was set at p < 0.05. Bivariate comparison between groups was done using t test, Mann Whitney U test and X2 test. Analysis was conducted at lesion level based on intention to treat approach. Lesions in children lost to follow up and surfaces that were restored or extracted by 6 months were considered active. The effect of the interventions on caries arrest (yes/ no) was assessed in bivariate analysis using Pearson Chi-Square test, followed by multi-level multiple binary logistic regression analysis to account for clustering of teeth in children and control for confounders: child sex and age, mother’s education, baseline lesion severity (moderate lesions- ICDAS codes 3 or 4 and advanced lesions- ICDAS codes 5 or 6), sugar score, dental visits, toothbrushing frequency, and plaque index. dmfs score was removed due to collinearity with ICDAS codes. Children were included as random effect variables. The interaction between the intervention and baseline lesion severity was assessed by calculating the interaction p value. Adjusted odds ratios (AORs), p value and 95% confidence intervals (CIs) overall and per subgroup were calculated.

## Results

The study included 1606 lesions in 220 children. SDF + NaF was applied to 749 lesions in 110 children and SDF was applied to 857 lesion in 110 children. The loss to follow up in SDF + NaF and SDF groups was 10% and 9% as shown in Fig. 1. Table 1 shows that the median (IQR) age was 48 (9) months and 50.4% were males with no significant differences between groups (p > 0.05). The mothers in the SDF + NaF group were significantly less educated (p = 0.002) and less children visited the dentist last year (p = 0.04) than in the SDF group. Also, the children in the SDF + NaF group had significantly lower dmfs score (p = 0.03) and plaque index score (p = 0.02) than the SDF group.

**Table 1:** Socio-demographic characteristics, oral health behaviors and clinical features in children included in the study at baseline (n = 220)

Variables		Groups		P value
		SDF + NaF n = 110	SDF n = 110	
Child age in months	Median (IQR)	48 (6)	48 (11)	0.06
Child sex	Male: n (%)	56 (50.9%)	55 (50%)	0.89
	Female: n (%)	54 (49.1%)	55 (50%)	
Mother’s education	Less than high school: n (%)	72 (65.5%)	49 (44.5%)	0.002
	High school and more: n (%)	38 (34.5%)	61 (55.5%)	
Sugar score	Median (IQR)	7.0 (1.3)	7.0 (2.0)	0.20
Dental visits in the last year	Yes: n (%)	44 (40.0%)	59 (53.6%)	0.04
	No: n (%)	66 (60.0%)	51 (46.4%)	
Toothbrushing once or more daily	Yes: n (%)	29 (26.4%)	38 (34.5%)	0.19
	No: n (%)	81 (73.6%)	72 (65.5%)	
dmfs score	Median (IQR)	6 (14)	10 (11)	0.03
Number of all included lesions	Median (IQR)	6 (5.3)	6.5 (7)	0.14
Number of moderate lesions	Mean ± SD	1.63 ± 1.56	1.66 ± 1.78	0.87
Number of advanced lesions	Mean ± SD	5.18 ± 5.28	6.10 ± 5.38	0.20
Plaque index	Median (IQR)	1.33 (0.67)	1.50 (0.55)	0.02

**Fig. 2:**
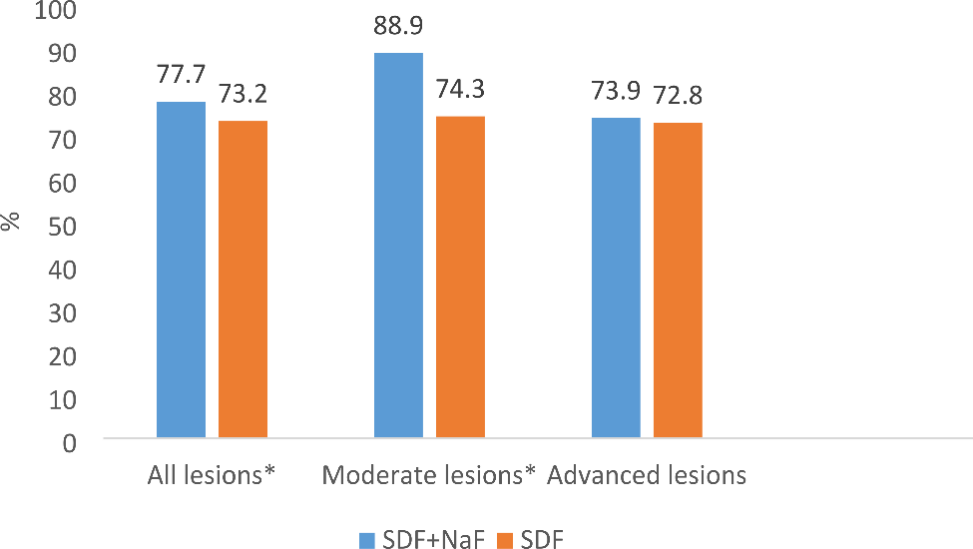
Lesion arrest rates in the study groups by baseline lesion severity (*: p < 0.05)

Figure 2 shows that the percentage of arrested lesions in the SDF + NaF group was significantly higher than the SDF group in all lesions (77.7% and 73.2%, p = 0.04) and in moderate lesions (88.9% and 74.3%, p < 0.001) but not in advanced lesions (73.9% and 72.8%, p = 0.37).

The multi-level multivariable logistic regression model in Table 2 showed that SDF + NaF had significantly higher odds of arresting ECC (AOR = 2.12, 95% CI: 1.08, 4.16, p = 0.03). There were no differences in arrest rates by child’s age or sex, mother’s educational level, sugar score, brushing frequency, dental visits, plaque index, or baseline lesion severity (p > 0.05).

**Table 2:** Multivariable multilevel logistic regression model for factors affecting ECC arrest after 6 months

Explanatory variables		AOR (95% CI)	P value
**Sociodemographic background**			
Child age in months		0.96 (0.92,1.00)	0.06
Child sex	Male	1.43 (0.75,2.74)	0.28
	Female^a^	1.00	
Mother’s educational level	High school and higher	1.13 (0.58,2.18)	0.72
	Less than high school^a^	1.00	
**Oral health behaviors**			
Sugar score		1.24 (0.96,1.60)	0.11
Toothbrushing once or more daily	Yes	1.70 (0.83,3.49)	0.15
	No^a^	1.00	
Dental visits last year	Yes	0.88 (0.46,1.68)	0.71
	No^a^	1.00	
**Clinical characteristics**			
Plaque index		1.03 (0.54,1.96)	0.94
Baseline caries severity	Advanced (ICDAS 5/6) lesions	0.68 (0.44,1.03)	0.07
	Moderate (ICDAS 3/4) lesions^a^	1.00	
Arm	SDF + NaF	2.12 (1.08,4.16)	0.03
	SDF^a^	1.00	

AOR, adjusted odds ratio; CI, confidence interval

^a^reference category

Table 3 shows that there was a significant interaction between the type of intervention and baseline lesion severity (p = 0.03). In moderate lesions, SDF + NaF had significantly greater arrest effect than SDF alone (AOR = 4.10, 95% CI: 1.53, 10.98, p = 0.005). In severe lesions, SDF + NaF had greater effect than SDF alone (AOR = 1.92, 95% CI: 0.93, 4.00), with no significant difference (p = 0.08).

**Table 3:** Multilevel multivariable binary logistic regression of the effect of SDF + NaF compared to SDF on caries arrest according to baseline lesion severity

Lesion severity at baseline	AOR (95%CI)	P value
Moderate lesions	4.10 (1.53, 10.98)	0.005
Advanced lesions	1.92 (0.93, 4.00)	0.08

The models were adjusted for child sex, child age, mother’s education, sugar score, dental visit in the last year, tooth brushing frequency, and plaque index.

P for the interaction between baseline lesion severity and intervention type = 0.03.

AOR: Adjusted odds ratio, CI: Confidence interval.

In the SDF + NaF group, more parents were satisfied with the child’s appearance than the SDF group (84.5% and 78.2%) with no significant difference between groups (p = 0.23). All carious lesions were stained black after SDF application in both groups. There was no tooth pain, gum pain, swelling, signs or symptoms of systemic toxicity including nausea or vomiting. Gum blanching was observed in 17 (15.4%) children in the SDF only group.

## Discussion

The study showed that combining SDF + NaF produced significantly higher arrest rates for all lesions and for moderate lesions. The superior effect of SDF + NaF was not observed in the advanced lesions that were cavitated at baseline and the difference between groups was not significant. Although more parents were satisfied with their children’s appearance in the SDF + NaF group, the difference was not statistically significant, and staining was present in both groups. No adverse events or systemic manifestations were reported in both groups and transient gum blanching was observed in few children in the SDF group. The findings of the study support the rejection of the null hypothesis indicating that there is a difference between the two fluoride regimens in ECC arrest.

The limitations of this study include the follow-up of 6 months which can be extended to longer follow-up periods in future studies to confirm the present conclusions. Also, some lesions might have been misdiagnosed since no radiographic examination was used because of the field conditions. Nonetheless, the study has several strengths including the large number of lesions which produced precise estimates with narrow confidence intervals. The exception for this was the estimate of the odds ratio for the arrest of moderate lesions which was imprecise because of the low number of this type of lesions per child. This reflects the distribution of lesions with different levels of severity in the study population with greater representation of advanced than moderate lesions. Another strength is the use of multilevel analysis to accommodate the clustering of teeth within children. Also, the community setting where the study was conducted supports the generalizability of findings to those with the greatest need of minimally invasive dental care.

The study findings have implication for the non-invasive management of ECC. For young children with lesions of different levels of severity, combing SDF and NaF may arrest more lesions while for patients with advanced lesions, using only SDF may be adequate. These finding are useful in low- and middle-income countries like Egypt where resources are limited and where material cost and availability may affect the decision to use minimally invasive dental care. However, cost effectiveness evaluation of both interventions is needed to inform recommendations. The study adds to the knowledge base by directly comparing the two fluoride regimens and providing evidence on their relative impact on lesions with different severities thus offering clinicians more options to manage ECC non-invasively.

The study also guides policy makers in planning and implementing prevention programs to reduce the burden of ECC in underserved areas with high disease burden.

The present findings show a stronger effect of SDF + NaF than SDF in arresting ECC lesions. Direct comparison with previous studies is difficult due to differences in the choice of agents, as studies combining SDF and NaF are scarce, as well as differences in frequencies of application and follow up periods. One study [16] assessed the efficacy of 38% SDF + 5% NaF varnish in arresting cavitated caries in primary teeth. This was a pilot feasibility study of 40 children less than 72 months and no control group. The investigators used a different protocol to apply the agents than the one used in the present study. They rinsed the lesions with water or wiped them with wet gauze after applying the SDF then applied NaF varnish once followed by assessing caries arrest after 4 months.

The authors reported an arrest of 74.1% of lesions, similar to the 77.7% in our study. Another trial registered in the clinicaltrials.gov registry (#NCT03480516) has been conducted in Thailand on school children aged 6–7 years old with active dentin caries lesions in primary canines and molars. The study aimed to assess caries arrest after applying 38% SDF and 5% NaF varnish every 6 months, but no results have been published yet.

The reason for adding NaF to SDF in the present study was to improve the caries arrest potential of SDF. Previous studies assessed the possibility of reducing SDF staining by adding potassium iodide (KI) with concomitant evaluation of caries arrest. One study reported that when KI was added to Riva Star SDF (Riva Star, SDI Inc., Australia), 82% of advanced caries lesions in the primary molars of 6–9-year-old children [36] were arrested after 6 months. Another study [37] reported higher odds ratio of arresting active caries in primary teeth of school children when Rivastar 38% + KI were used than SDF alone (AOR 1.23; CI 1.06–1.43). Thus, the effect of SDF may be increased by combining it with other agents including NaF as shown in the present study or KI in previous studies.

The study findings showed a stronger effect of SDF + NaF than SDF alone in moderate lesions rather than a weaker effect on advanced lesions where the percentage of SDF + NaF arrested moderate lesions was the highest among all subgroups possibly because of the effect of NaF on enamel/ moderate lesions [7, 15]. In advanced lesions, there is more dentine than enamel per unit area compared to moderate lesions. This might have given NaF less possibilities to exert its caries preventive effect.

Our findings are also consistent with studies showing the relative advantage of NaF varnish in remineralizing enamel (ICDAS code 3) lesions [7, 15] which are among the moderate lesions included in the present study.

The greater arrest effect of SDF on dentine than enamel caries was documented in previous studies [7, 12, 27] where SDF had greater effect than NaF on arresting dentin caries in primary teeth (20.5% and 12.3%) [27]. Our findings, however, disagree with a study reporting no superior effect for NaF over SDF (58.8% and 59.1%) in arresting enamel caries in primary teeth [38]. The reason for the difference between the studies may be due to using NaF alone without combining it with SDF and potential bias in assessing the outcome by a single examiner who identified the SDF cases by the black staining. However, the possibility exists that the greater caries preventive effect of SDF + NaF may be due to the varnish of NaF covering the SDF, ensuring its adherence to the tooth surface for a longer time and delaying its wash away from the oral cavity. Future studies are recommended to disentangle the effect of coverage with varnish from the fluoride action by including a group where SDF is covered with sham varnish with no active ingredient.

Parents’ satisfaction with the appearance of their children’s teeth was high although teeth were stained. This can be attributed to the rural setting where the study was conducted where cultural norms and limited availability of services might have affected their acceptance of staining. These findings are consistent with studies conducted in China were parental satisfaction ranged from 65.7 to 70.3% [27, 29]. However, care should be taken when generalizing these findings to other cultures and settings where dental esthetics is a concern.

Further studies are needed in a clinical setting with radiographic assessment of lesion progress among children of young age and those with special needs where the intervention may be useful. This would complement the findings of our study which targets children from disadvantaged backgrounds in field conditions. Also, Further research is needed to assess the effect of both regimens on caries increment and on reducing the number of new lesions affected by caries beyond the lesions on which the agents are applied, for longer periods as well as cost effectiveness of both interventions. This information would help guide the decision to combine SDF and NaF to prevent ECC depending on the stage of lesion progress.

## Conclusion

The study showed that combining SDF and NaF induced greater arrest of ECC lesions than SDF alone and this difference was significant in moderate but not advanced lesions. Parental satisfaction with children’s appearance did not differ between groups. These findings have implications for the non-invasive management of ECC in field conditions to meet the dental care needs of disadvantaged children in rural communities.

## Data Availability

The datasets used and/or analysed during the current study are available from the corresponding author on reasonable request.

## List of abbreviations

ECC: Early Childhood Caries
NaF: Sodium Fluoride
SDF: Silver Diamine Fluoride
ICDAS: International Caries Detection and Assessment System
LAA criteria: lesion activity assessment
dmfs: decayed, missing and filled surfaces in primary teeth
CPI: Community Periodontal Index

## References

[1] Dentistry AAOP. Policy on early childhood caries (ECC): classifications, consequences, and preventive strategies. Ref Man Pediatr Dentistry 2016(1942–5473 (Electronic)). 27931420

[2] Colak H. Dülgergil Ct Fau - Dalli M, Dalli M Fau - Hamidi MM, Hamidi MM: Early childhood caries update: A review of causes, diagnoses, and treatments. 2013(0976–9668 (Print)). 10.4103/0976-9668.107257 PMC3633299 23633832

[3] Bernabe E, Hernandez WMCR, Bailey J, Abreu LG, Alipour V, Amini S, Arabloo J, Arefi Z, Arora A, Ayanore MA et al. Global, Regional, and National Levels and Trends in Burden of Oral Conditions from 1990 to 2017: A Systematic Analysis for the Global Burden of Disease 2017 Study. 2020(1544 – 0591 (Electronic)). 10.1177/0022034520908533 PMC7088322 32122215

[4] Zaror CA-O, Matamala-Santander A, Ferrer M, Rivera-Mendoza F, Espinoza-Espinoza G, Martínez-Zapata MJ. Impact of early childhood caries on oral health-related quality of life: a systematic review and meta-analysis. (1601–5037 (Electronic)). 10.1111/idh.12494 33825317

[5] Oubenyahya HA-O, Bouhabba NA-O. General anesthesia in the management of early childhood caries: an overview. (2383–9309 (Print)). 10.17245/jdapm.2019.19.6.313 PMC6946837 31942447

[6] Thomson WA-O. Public Health Aspects of Paediatric Dental Treatment under General Anaesthetic. LID – 10.3390/dj4020020 [doi] LID – 20. (2304–6767 (Electronic)). 10.3390/dj4020020 PMC5851258 29563462

[7] Gao SS, Zhang S, Mei ML, Lo EC, Chu CH (2016). Caries remineralisation and arresting effect in children by professionally applied fluoride treatment - a systematic review. BMC Oral Health.

[8] Chu CH, Lo E. Uses of sodium fluoride varnish in dental practice. 2008(0158–1570 (Print)). 19728633

[9] Mei ML, Ito L, Cao Y, Lo EC, Li QL, Chu CH. An ex vivo study of arrested primary teeth caries with silver diamine fluoride therapy. 2014(1879-176X (Electronic)). 10.1016/j.jdent.2013.12.007 24373856

[10] Mei ML, Ito L, Fau - Cao Y, Cao Y, Fau - Li QL, Li Ql Fau - Lo ECM, Lo Ec Fau -, Chu CH, Chu CH. Inhibitory effect of silver diamine fluoride on dentine demineralisation and collagen degradation. (1879-176X (Electronic)). 10.1016/j.jdent.2013.06.009 23810851

[11] Seifo N, Cassie H, Radford JR, Innes NA-O. Silver diamine fluoride for managing carious lesions: an umbrella review. 2019(1472–6831 (Electronic)). 10.1186/s12903-019-0830-5 PMC6626340 31299955

[12] Duangthip D, Wong MCM, Chu CH, Lo ECM (2018). Caries arrest by topical fluorides in preschool children: 30-month results. J Dent.

[13] Chibinski AC, Wambier Lm Fau - Feltrin J, Feltrin J, Fau - Loguercio AD. Loguercio Ad Fau - Wambier DS, Wambier ds Fau - Reis A, Reis A: silver diamine fluoride has Efficacy in Controlling Caries Progression in primary teeth: a systematic review and Meta-analysis. 2017(1421-976X (Electronic)). 10.1159/000478668 28972954

[14] Clemens J, Gold J, Chaffin J. Effect and acceptance of silver diamine fluoride treatment on dental caries in primary teeth. 2018(1752–7325 (Electronic)). 10.1111/jphd.12241 28749529

[15] Crystal YO, Niederman R. Silver Diamine Fluoride Treatment Considerations in Children’s Caries Management. 2016(1942–5473 (Electronic)). PMC5347149 28281949

[16] Sihra R, Schroth RJ, Bertone M, Martin H, Patterson B, Mittermuller B-A, Lee V, Patterson B, Moffatt ME, Klus B (2020). The effectiveness of Silver Diamine Fluoride and Fluoride Varnish in Arresting caries in Young Children and Associated oral health-related quality of life. J Can Dent Assoc.

[17] Fadl AE, Abdel Fattah R, Ezz M (2019). Assessing the prevalence of early childhood caries and the associated determinants in a group of preschool children: results from a national oral health survey in Egypt. Egypt Dent J.

[18] El Tantawi M, Folayan MO, Mehaina M, Vukovic A, Castillo JL, Gaffar BO, Arheiam A, Al-Batayneh OB, Kemoli AM, Schroth RJ et al. Prevalence and Data Availability of Early Childhood Caries in 193 United Nations Countries, 2007–2017. 2018(1541-0048 (Electronic)). 10.2105/AJPH.2018.304466 PMC6050821 29927650

[19] Population Estimates By Governorate. (Urban /Rural) [https://www.capmas.gov.eg/Pages/StaticPages.aspx?page_id=5035].

[20] Ismail AI, Sohn W, Fau - Tellez M, Tellez M, Fau - Amaya A, Amaya A, Fau - Sen A, Sen A, Fau - Hasson H, Hasson H. Fau - Pitts NB, Pitts NB: the International Caries Detection and Assessment System (ICDAS): an integrated system for measuring dental caries. 2007(0301–5661 (Print)). 10.1111/j.1600-0528.2007.00347.x 17518963

[21] Association WM. World Medical Association Declaration of Helsinki: ethical principles for medical research involving human subjects. 2013(1538–3598 (Electronic)). 10.1001/jama.2013.281053 24141714

[22] Schulz KF, Altman DG, Moher D, the CG (2010). CONSORT 2010 Statement: updated guidelines for reporting parallel group randomised trials. BMC Med.

[23] Vaikuntam J. Fluoride varnishes: should we be using them? Pediatr Dent 2000(0164–1263 (Print)):4. 11132514

[24] Ekstrand KR, Martignon S, Fau - Ricketts DJN, Ricketts Dj Fau -, Qvist V, Qvist V. Detection and activity assessment of primary coronal caries lesions: a methodologic study. Oper Dent 2007(0361–7734 (Print)). 10.2341/06-63 17555173

[25] (ICCMS) International Caries Classification and Management System, Resources. [https://www.iccms-web.com/].

[26] McHugh ML. Interrater reliability: the kappa statistic. Biochem Med (Zagreb) 2012(1330 – 0962 (Print)). PMC3900052 23092060

[27] Mabangkhru S, Duangthip D, Chu CH, Phonghanyudh A, Jirarattanasopha V. A randomized clinical trial to arrest dentin caries in young children using silver diamine fluoride. J Dent 2020(1879-176X (Electronic)). 10.1016/j.jdent.2020.103375 32428523

[28] Kittiprawong R, Kitsahawong K, Pitiphat W, Dasanayake A, Pungchanchaikul P. Parent-child satisfaction and Safety of Silver Diamine Fluoride and Fluoride Varnish Treatment. Int J Oral Health 2018.

[29] Duangthip D, Fung MA-O, Wong MCM, Chu CA-O, Lo ECM. Adverse effects of Silver Diamine Fluoride Treatment among Preschool Children. J Dent Res 2018(1544 – 0591 (Electronic)). 10.1177/0022034517746678 29237131

[30] Silness J, Fau - Loe H, Loe H. PERIODONTAL DISEASE IN PREGNANCY. II. CORRELATION BETWEEN ORAL HYGIENE AND PERIODONTAL CONDTION. Acta Odontol Scand 1964(0001–6357 (Print)). 10.3109/00016356408993968 14158464

[31] Petersen PE, Baez RJ, World Health O (2013). Oral health surveys: basic methods.

[32] Bokhari AM, Quadri MA-O. What factors contribute to the self-reported oral health status of arab adolescents? An assessment using a validated Arabic-WHO tool for child oral health (A-OHAT). BMC Oral Health 2020(1472–6831 (Electronic)). 10.1186/s12903-020-1018-8 PMC6986000 31992291

[33] Fung MHT, Duangthip D, Wong MCM, Lo ECM, Chu CH. Arresting dentine caries with different concentration and periodicity of silver diamine fluoride. JDR Clin Translational Res 2016(2380 – 0844). 10.1177/2380084416649150 PMC5615850 28989974

[34] Faul F, Erdfelder E, Fau - Lang A-G, Lang Ag Fau - Buchner A, Buchner A. G*Power 3: a flexible statistical power analysis program for the social, behavioral, and biomedical sciences. Behav Res Methods 2007(1554-351X (Print)). 10.3758/bf03193146 17695343

[35] Research Randomizer(Version. 4.0) [Computer software] [https://www.randomizer.org].

[36] Prakash DKM, Vinay C, Uloopi KS, RojaRamya KS, Penmatsa C, Chandana N. Evaluation of caries arresting potential of silver diamine fluoride and sodium fluoride varnish in primary molars: a randomized controlled trial. J Indian Soc Pedod Prev Dentistry 2022, 40(4). 10.4103/jisppd.jisppd_239_22 36861553

[37] Turton B, Horn R, Durward C. Caries arrest and lesion appearance using two different silver fluoride therapies with and without potassium iodide: 6-month results. Heliyon 2020(2405–8440 (Print)). 10.1016/j.heliyon.2020.e04287 PMC7369606 32715116

[38] Phonghanyudh A, Duangthip DA-O, Mabangkhru S, Jirarattanasopha VA-O. Is silver diamine fluoride effective in arresting Enamel caries? A Randomized Clinical Trial. LID – 10.3390/ijerph19158992 [doi] LID – 8992. Int J Environ Res Public Health 2022(1660–4601 (Electronic)). 10.3390/ijerph19158992 PMC9331268 35897363

